# Orotracheal Intubation Using the Retromolar Space: A Reliable Alternative Intubation Approach to Prevent Dental Injury

**DOI:** 10.1155/2016/3529415

**Published:** 2016-12-25

**Authors:** Linh T. Nguyen, Sudip D. Thakar, Angela T. Truong, Dam-Thuy Truong

**Affiliations:** ^1^UT MD Anderson Cancer Center, Anesthesiology & Perioperative Medicine, 1400 Holcombe Blvd, Unit 409, Houston, TX 77030, USA; ^2^Baylor College of Medicine, 1 Baylor Plaza, Houston, TX 77030, USA

## Abstract

Despite recent advances in airway management, perianesthetic dental injury remains one of the most common anesthesia-related adverse events and cause for malpractice litigation against anesthesia providers. Recommended precautions for prevention of dental damage may not always be effective because these techniques involve contact and pressure exerted on vulnerable teeth. We describe a novel approach using the retromolar space to insert a flexible fiberscope for tracheal tube placement as a reliable method to achieve atraumatic tracheal intubation. Written consent for publication has been obtained from the patient.

## 1. Introduction

Perianesthetic dental injuries account for the largest number of anesthesia-related adverse events and the major cause for malpractice litigation against anesthesia providers according to closed claims data bases [1]. Recent prevention advances in intubation devices and prevention strategies using protective dental guards, videolaryngoscopes, and flexible fiberoptic bronchoscopes with intubating airways have not been effective in decreasing the incidence of dental injuries. As an effective means to prevent dental injury, we propose using the retromolar space to insert a flexible fiberoptic bronchoscope to minimize contact with the vulnerable teeth during intubation.

## 2. Case Description

**Figure 1:**
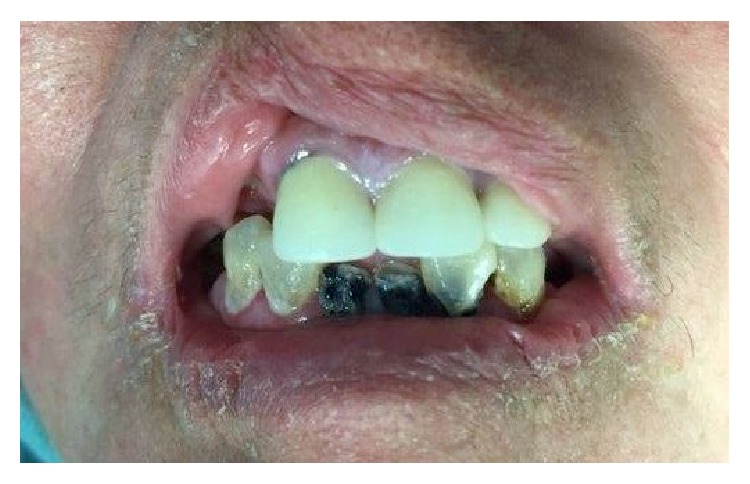
Preoperative airway examination showing extremely poor dental condition.

A 75-year-old female with metastatic renal cell carcinoma to the left proximal femur presented for resection of the femur under general anesthesia. Her past medical history included hypertension and well controlled diabetes mellitus. Preoperative airway assessment showed adequate mouth opening and normal neck range of motion. Dentition was in very poor condition. Examination of her oral cavity revealed poor oral hygiene, severe periodontal disease, prominent restored upper incisors with veneers, and several broken lower teeth (Figure 1). Using the Miller tooth mobility classification, several teeth were classified as class I (greater mobility than normal physiologic) and class II (tooth mobility is 1 mm or more laterally, but not in a vertical direction) [2]. Both nasal passages were stenosed due to prior nasal fractures.

**Figure 2:**
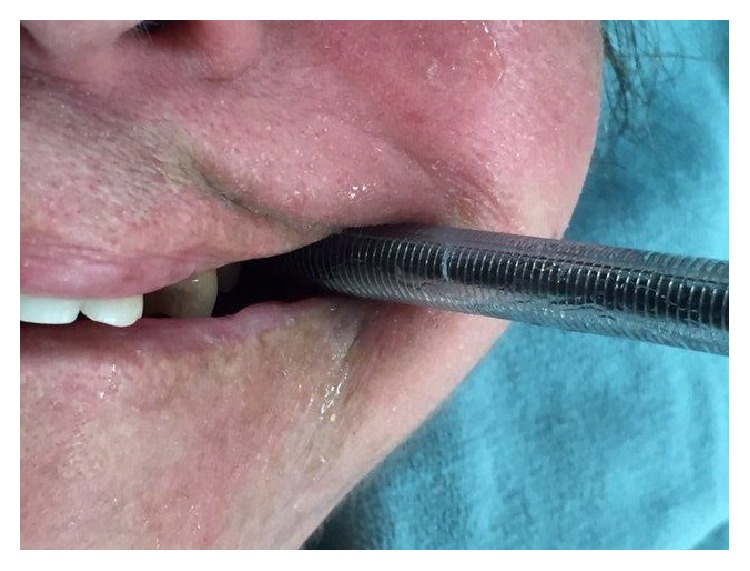
Postretromolar intubation showing tracheal tube in contact only with the last molars.

In the operating room, standard monitors were applied and the patient was preoxygenated with 100% oxygen. Intravenous propofol 60 mg and fentanyl 200 mcg were administered. Following adequate bag and mask ventilation with 100% oxygen, intravenous rocuronium 50 mg was given. A well-lubricated 36 French nasopharyngeal airway was inserted into the left retromolar space to estimate the size of the space, provide adequate lubrication, and create a memory path for subsequent tracheal tube insertion. A 3.5 mm flexible fiberoptic bronchoscope armed with a 6.5 mm wired reinforced tracheal tube was inserted into the left corner of the mouth, advanced to the left retromolar space and guided through the vocal cords. Once the scope was in the trachea, the tube was then advanced over the scope for tracheal intubation (Figure 2). During this procedure, there was direct contact only to the posterolateral surfaces of the last molars by the fiberscope and the tracheal tube. After intubation, all teeth were intact. At the completion of surgery, the tracheal tube was removed. All the teeth were accounted for and no dental damage was detected.

## 3. Discussion

Orotracheal intubation is commonly performed to secure and protect the airway in patients requiring general anesthesia for surgical procedures. Even in experienced hands, intraoral manipulation may cause injuries to the teeth with considerable functional, cosmetic, psychological, and financial consequences. The overall incidence of anesthesia-related dental injuries is estimated to be between 0.06% and 12% [2]. Similar to other iatrogenic adverse events, perioperative dental injuries are subject to forensic liability. In fact, dental injuries are the most frequent cause for malpractice litigation against anesthesia providers [3].

The risk factors for dental injuries include poor dentition and difficult intubation due to prominent upper incisors, limited mouth opening, and short bulky neck. The severity of injury ranges from minimal enamel damage to crown or root fracture and complete tooth avulsion. An unrecognized dislodged tooth may be inhaled into the lung, causing pneumonias, abscesses, and bronchiectasis requiring invasive procedures to retrieve it.

The maxillary incisors are most commonly injured by a laryngoscope blade, especially when used as a fulcrum to expose the epiglottis. Considering the magnitude of the problem, various strategies for prevention of dental injury have been recommended. Preoperative assessment of the airway and preexisting dental abnormalities and factors which may increase dental fragility should be carefully documented. Tooth guards or occlusal gutters may be valuable in protecting teeth with veneers and porcelain restorations during intubation. Unfortunately, in cases of already loose teeth, their insertion and removal may in fact directly cause teeth avulsion.

During airway management the placement and removal of oral airways and supraglottic devices such as laryngeal mask airways may cause injury to precarious teeth. Nasal intubation using a rigid direct laryngoscope and Magill forceps also carries the risk of injury to vulnerable teeth. Use of videolaryngoscopes such as Airtraq, Glidescope, and C-MAC® (Karl Storz Endoscopy America, Stafford, TX) may be beneficial in preventing dental trauma by reducing the need to lean against the upper incisors. If the teeth are already loose, however, the intraoral manipulation required for the insertion and removal of these hard steel blades may also directly fracture or dislodge the vulnerable teeth.

The flexible fiberoptic bronchoscope may offer another option to decrease dental injuries. Unfortunately, the conventional intraoral approach using intubating airways such as the Ovassapian airway may directly cause dental injuries during the insertion and removal of these devices due to direct contact and pressure against vulnerable teeth.

Thus, current strategies for prevention of dental injuries have not been effective or reliable. Furthermore, the level of training of the operator does not influence the occurrence of dental injuries related to oral intubations [4]. Flexible fiberoptic nasal intubation is effective in preventing dental injury. However, the nasal route is contraindicated in patients with intranasal pathology, bleeding diathesis, and basal skull fractures. It is not an intubation route appropriate for surgery involving the nose, paranasal sinuses, nasolacrimal ducts, or transphenoidal hypophysectomy. Furthermore, nasotracheal intubation may cause serious complications including trauma to nasal structures and epistaxis [5]. In this case, nasotracheal intubation was not performed due to the existence of bilateral nasal stenosis and risk of infection in an immunocompromised cancer patient.

Therefore, an alternative approach is needed. In contrast to the previously mentioned techniques which apply direct forces on the vulnerable teeth, flexible fiberoptic intubation through the retromolar space is a much more effective and reliable technique to prevent dental injuries because it involves minimal contact with the teeth.

Bounded anteriorly by the last erupted molar, posteriorly by the ramus of the mandible, superiorly by the maxillary tuberosity, and inferiorly by the retromolar trigone, the retromolar space has been used for flexible fiberoptic oral intubation in patients with severe trismus in whom the reduced interincisor distance does not allow for placement of a rigid laryngoscope or a tracheal tube between the teeth [6].

Retromolar space dimensions using dental pantomograms have reported a mean height of 17.9 mm for the right space and 18.1 mm for the left space. The reported mean width was 17.5 mm for the right space and 16.51 mm for the left space [7]. These measurements show that the retromolar space is large enough to readily fit an 8.0 mm tracheal tube, which has an outer diameter of 10.8 mm. Because of the existence of two retromolar spaces, right and left, even in the presence of loose teeth or a retromolar trigome lesion on one side, the uninvolved contralateral side can be safely used for tube placement. The skills required to perform retromolar flexible fiberoptic intubation are essentially the same needed for conventional fiberoptic oral or nasal intubations. An operator with expertise in fiberoptic bronchoscopy techniques can master this new approach without difficulty. The main advantage of this approach is that, by advancing the fiberoptic scope into the retromolar space into the pharyngeal cavity, the intraoral structures, including the tongue and intraoral secretions, are bypassed. Based on teaching residents and fellows, this technique is surprisingly easy to learn. Competence can be reasonably achieved after 20–30 successful retromolar intubations.

The retromolar space provides a valuable and safe point of entry far away from vulnerable teeth for tracheal intubation in patients with poor dentition because, throughout this procedure, the only teeth in direct contact with the bronchoscope and the tube are the last molars.
